# Proof-of-Concept Study on an Automatic Computational System in Detecting and Classifying Occlusal Caries Lesions from Smartphone Color Images of Unrestored Extracted Teeth

**DOI:** 10.3390/diagnostics11071136

**Published:** 2021-06-22

**Authors:** Duc Long Duong, Quoc Duy Nam Nguyen, Minh Son Tong, Manh Tuan Vu, Joseph Dy Lim, Rong Fu Kuo

**Affiliations:** 1 Department of Biomedical Engineering, National Cheng Kung University, Dasyue Rd, Tainan 701, Taiwan; namduy1012@gmail.com (Q.D.N.N.); wankuo@gmail.com (R.F.K.); 2 School of Odonto-Stomatology, Hanoi Medical University, Ton That Tung St, Hanoi City 10000, Vietnam; tongminhson@hmu.edu.vn (M.S.T.); manhtuan@hmu.edu.vn (M.T.V.); 3 Center of Dentistry, COAHS, University of Makati, J.P. Rizal Ext, Makati, Metro Manila 1215, Philippines; josephdlim@yahoo.com; 4 Medical Device Innovation Center, National Cheng Kung University, Shengli Rd, Tainan 704, Taiwan

**Keywords:** caries detection, occlusal caries, feature selection, machine learning, support vector machine, digital imaging

## Abstract

Dental caries has been considered the heaviest worldwide oral health burden affecting a significant proportion of the population. To prevent dental caries, an appropriate and accurate early detection method is demanded. This proof-of-concept study aims to develop a two-stage computational system that can detect early occlusal caries from smartphone color images of unrestored extracted teeth according to modified International Caries Detection and Assessment System (ICDAS) criteria (3 classes: Code 0; Code 1–2; Code 3–6): in the first stage, carious lesion areas were identified and extracted from sound tooth regions. Then, five characteristic features of these areas were intendedly selected and calculated to be inputted into the classification stage, where five classifiers (Support Vector Machine, Random Forests, K-Nearest Neighbors, Gradient Boosted Tree, Logistic Regression) were evaluated to determine the best one among them. On a set of 587 smartphone images of extracted teeth, our system achieved accuracy, sensitivity, and specificity that were 87.39%, 89.88%, and 68.86% in the detection stage when compared to modified visual and image-based ICDAS criteria. For the classification stage, the Support Vector Machine model was recorded as the best model with accuracy, sensitivity, and specificity at 88.76%, 92.31%, and 85.21%. As the first step in developing the technology, our present findings confirm the feasibility of using smartphone color images to employ Artificial Intelligence algorithms in caries detection. To improve the performance of the proposed system, there is a need for further development in both in vitro and in vivo modeling. Besides that, an applicable system for accurately taking intra-oral images that can capture entire dental arches including the occlusal surfaces of premolars and molars also needs to be developed.

## 1. Introduction

Dental caries has been considered the heaviest worldwide oral health burden affecting a significant proportion of the population. More specifically, more than 10% of the world’s population was affected by permanent caries without pain, which became one of the eight major causes of chronic disease and injury [1]. It has been well documented that early detection of dental caries is an extremely desirable issue for strategies to prevent dental caries [2].

Conventionally, caries detection methods include visual-tactile examination and dental radiography. Dental radiography is an important tool that can be used for recognizing hidden carious lesions. However, in the long-term, dental X-rays also exposed some disadvantages such as: some types of conventional intra-oral X-ray systems (panoramic, wall-mounted periapical or bitewing radiographs) are immobile; they are hard to employ for dental public health investigation in rural areas with no radiology rooms; and the patients have to be subjected to radiation exposure from devices. In addition, X-ray images can typically display the presence of dental caries lesions in more severe stages with visible cavities while for diagnosis of early-stage caries, dentists are not able to detect the lesions from dental radiography, especially on buccal or lingual surfaces of teeth.

In clinical practice, the visual-tactile examination based on the experience of the dentists makes it become subjective. This variability in diagnosis leads to different caries management strategies, and not all of them achieve the expected result. So as to reduce the variability of dental caries diagnosis, several detection techniques have been developed based on optical characteristics of the lesions. These include Quantitative Light-Induced Fluorescence (QLF) [3], Photothermal Radiometry-Modulated Luminescence (PTR-LUM—“The Canary System”) [4,5], Laser-Induced Fluorescence (LIF) [6], Transillumination with Near-Infrared Light (NILT) [7], Fiber-optic Transillumination (FOTI) [8], Electrical Conductance (EC) [9], and digital photography analysis [10,11,12]. Among them, dental photography has shown its potentiality as a non-invasive and low-cost method in the detection of occlusal caries. Bottenberg and colleagues [10] reported that using photographic images to evaluate occlusal surfaces was not statistically different from scoring the extracted teeth according to the histological gold standard. Boye et al. [11] demonstrated that the assessments of the photographic images as a caries detection method had greater sensitivity than a visual examination on extracted permanent teeth with histology as the reference standard.

Furthermore, besides using the visual analysis method to detect caries from digital images, there have been numerous studies to investigate the feasibility of Artificial Intelligence (AI) technology to address this issue. Kositbowornchai et al. [13] designed a Learning Vector Quantization (LVQ) model using images from a charged-coupled device (CCD) camera and intra-oral digital radiography to diagnose artificial dental caries with tooth cross-sections as the gold standard. The sensitivity and specificity of dental caries detection by the CCD camera were 0.77 and 0.85, whereas they were 0.81 and 0.93 by digital radiography. Notwithstanding, this method has been assessed only to a limited extent since the system had only evaluated artificial carious lesions, which were totally dissimilar from natural carious lesions. Olsen et al. [14] proposed to employ the Directed Active Shape Modeling (DASM) algorithm to segment digital color images of drilled dental preparations for detecting damaged areas on occlusal surfaces. After feature extraction, the generated feature vectors were used to build a predictive model to identify pixels that represent damaged areas on tooth surfaces which were caused by caries, with the best accuracy being at 96.86%. The main drawback of this method was that the proposed system cannot detect caries in the early stage, which is visually non-cavitated (without any visible damage). Ghaedi et al. [15] proposed an automated dental caries detection and scoring system for optical images of tooth occlusal surface according to the ICDAS guidelines with accuracy, sensitivity, and specificity at 86.3%, 83%, and 91.7%. Berdouses et al. [16] presented a computer-aided automated methodology for the assessment of occlusal surfaces from photographic color images for caries detection and classification. When compared with visual assessment using ICDAS criteria, the accuracies of this method were 83% (7 classes) and 86% (3 classes) with sensitivity and specificity at 80% and 74%, respectively.

In the above-mentioned research, the pixel-based features were normally extracted from tooth images by the computational data analysis techniques and then were selected for use in classification algorithms. The selected features obviously are useful and informative to train the predictive models. However, in clinical settings, the dentists may not find those features helpful in diagnosing caries. To overcome these issues, the development of a new procedure to select and quantify the values of features based on characteristic features of caries properties is considered.

Evidently, previous studies were limited to analyzing images from digital cameras for caries detection. It is well known that Digital Single Lens Reflex (DSLR) cameras are recommended to use in dental photography [17]. The high-definition macro images taken by DSLR cameras have undoubtedly assisted dentists in clinical routine, but mobile devices also produce pictures with equally good quality due to advancements in technology development. Kohara et al. [18] concluded that smartphone images are feasible and accurate for distinguishing sound tooth surfaces from extensive caries lesions. One approach employed by Estai et al. [19] is to detect occlusal caries from photographs taken by a smartphone camera with an acceptable diagnostic performance compared to traditional face-to-face screening. Although using digital color images for caries detection has many challenges such as the difficulty of examining occlusal surfaces of posterior teeth from images in some cases, or that the resolution and quality of images can be impacted when a mirror is needed to photograph occlusal surfaces of posterior teeth, its potential in caries detection cannot be underestimated. To our knowledge, there are limited numbers of studies that have used smartphone color images as the input of the classification algorithms to identify and classify carious lesions into different categories.

The aims of this work are twofold: first, to present an identification methodology for carious lesion areas from smartphone color images. Second, the identified carious lesion areas are extracted from the sound tooth regions and then the values of characteristic features of these areas are quantified to use for early caries classification. Towards this direction, we use five classifiers: Support Vector Machine (SVM), Random Forests (RF), K-Nearest Neighbors (KNN), Gradient Boosted Tree (GBT), and Logistic Regression (LR) are employed to find the most accurate classifier among them.

## 2. Materials and Methods

**Figure 1:**
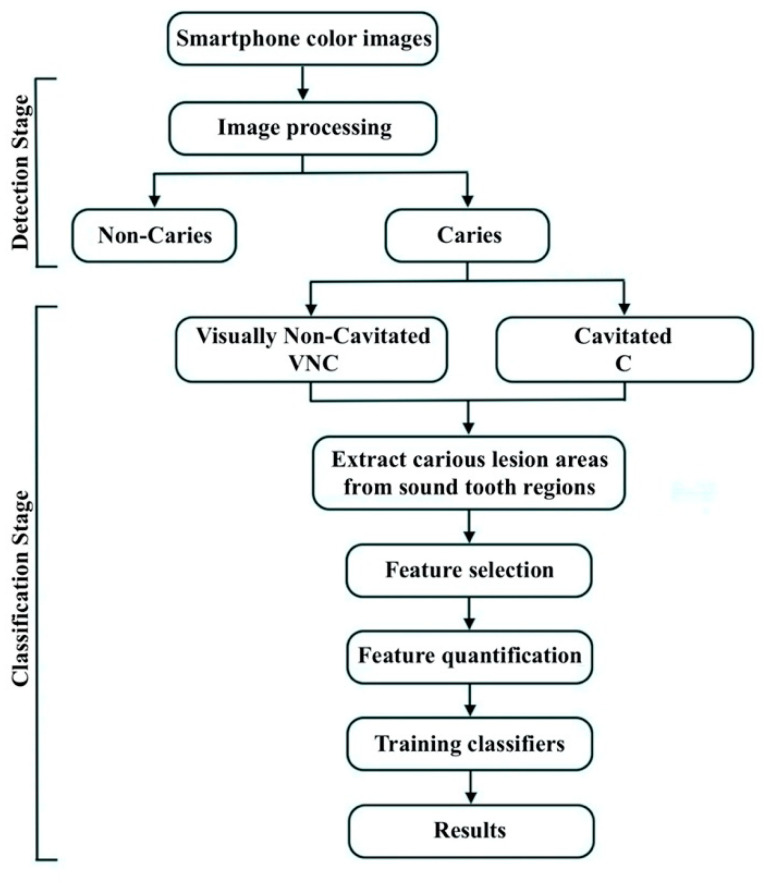
Schematic representation of the proposed system.

The overall workflow of the proposed system is illustrated in Figure 1.

### 2.1. Dataset

**Figure 2:**
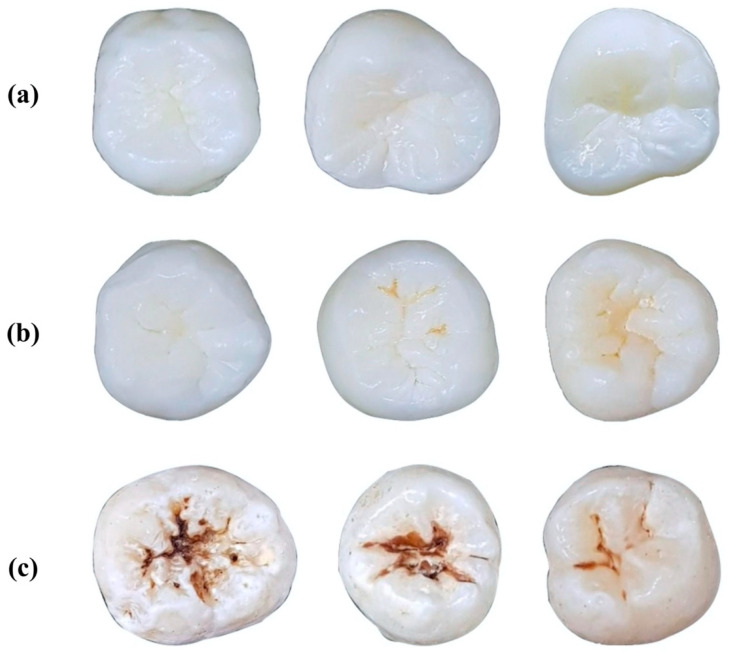
Examples of dataset. (**a**) No Surface Change class; (**b**) Visually Non-Cavitated class; (**c**) Cavitated class.

In this study, in order to classify dental caries, we referred to well-established International Caries Detection and Assessment System (ICDAS) criteria [20]. A total of 587 preprocessed smartphone color images of extracted molars and premolars were used in this study. These images were the same as those used in a previously published study by the same authors [21]. As mentioned in a previous study, due to the limited number for each ICDAS code, we grouped our dataset into 3 classes (see Figure 2): No Surface Change—NSC (Code 0), Visually Non-Cavitated—VNC (Code 1–2), Cavitated—C (Code 3–6). The dataset is shown in Table 1.

### 2.2. Caries Detection Stage

#### 2.2.1. Image Processing

For this stage, with the only purpose being to identify carious lesion areas from images, we merged VNC class and C class into the “*Caries*” category (ICDAS Code 1-6), while NSC class remained with the same number of images, but we named it the “*Non-Caries*” category (ICDAS Code 0). Then, the image processing algorithm was applied for both categories, “*Caries*” and “*Non-Caries*”. The OpenCV software package [22] was applied to perform the image processing procedure. The process is described as follows:

**Step 1.** ImageEnhance Module and Histogram equalization are applied to the whole dataset for contrast enhancement.

**Step 2.** Convert all color images to grayscale images (*gray_img*).

**Step 3.** Convert grayscale images to binary images (*binary_img*).

**Step 4.** Apply Contour Finding algorithm to find the largest boundary of tooth image in *binary_img*. Use information from accepted contour to create a *mask_binary*.

**Step 5.** The relative area outside the contour in the mask_binary is filled with the value 255 to create *thick_mask*.

**Step 6.** Tooth contour image (*tooth_contour_img*) = 255—*thick_mask*.

**Step 7.** Then, the image of the carious lesion area (*lesion_area_img*) is created: *lesion_area_img* = *tooth_contour_img*—*binary_img.*

**Step 8.** The Contour Finding algorithm is applied again for *lesion_area_img* to find the largest boundary of lesion and *mask_lesion* is created from the accepted contour.

**Step 9.** The relative area outside the contour in the *mask_lesion* is filled with the value 255.

**Step 10.** The relative area inside the contour in *mask_lesion* is replaced with the information of original image.

**Figure 3:**
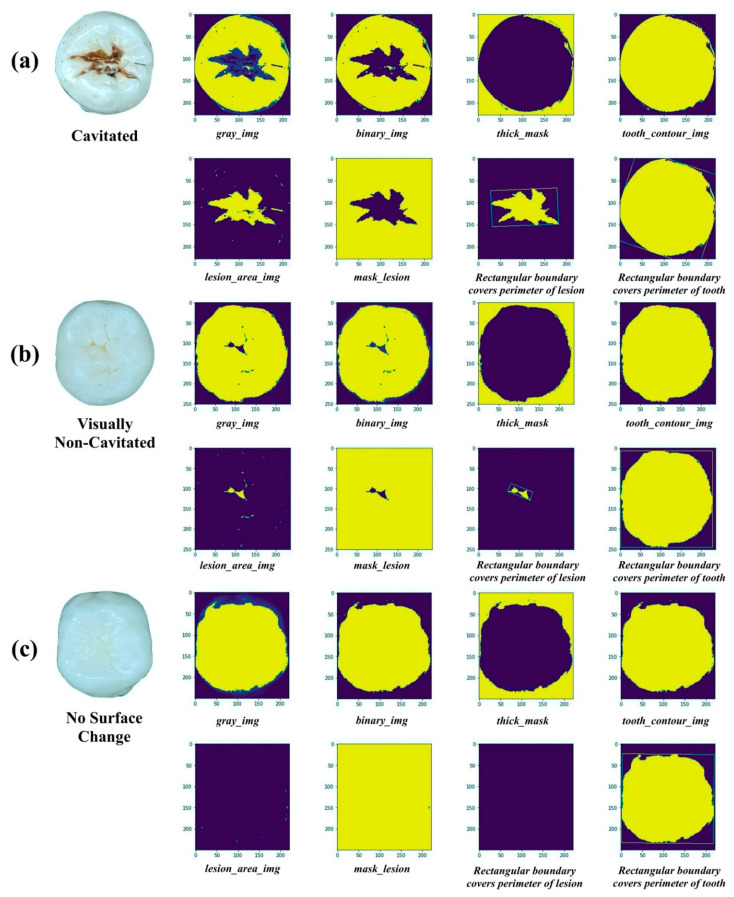
Proposed image processing method for caries detection. (**a**) Cavitated class; (**b**) Visually Non-Cavitated class; (**c**) No Surface Change class.

The performance of the proposed image processing method for caries detection with both “*Caries*” and “*Non-Caries*” categories is demonstrated in Figure 3. The post-processing image of a carious lesion area was correctly extracted and shown in Figure 3a with C class and in Figure 3b with VNC class, whereas, by the same process, the output image of “*Non-Caries*” category did not display any information on the presence of occlusal caries (see Figure 3c).

#### 2.2.2. Evaluation Process for Caries Detection Stage

**Figure 4:**
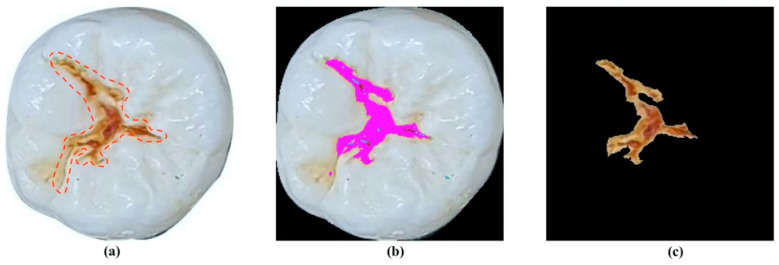
Example of results of caries detection approach. (**a**) Carious lesion area was identified by the experts; (**b**) carious lesion area was identified by proposed method; (**c**) extracted carious lesion area.

To clarify the performance of the proposed method, an evaluation process was established. Firstly, the entire dataset was analyzed by four dentists to identify and annotate the carious lesion areas from those images. The analyzed results of all dentists through images were reviewed to distinguish differences in evaluation. The final decision was made based on the inter-examiner agreements after discussion. Afterward, images of carious lesion areas, which were recognized by the proposed system, were projected on the images annotated by dentists to evaluate the overlap in regions between them. The proposed system was considered correct when the overlap in regions between two images was greater than 60% (see Figure 4).

### 2.3. Caries Classification Stage

#### 2.3.1. Feature Selection and Quantification

After carious lesion areas were detected, these areas were extracted from sound tooth regions. Next, according to the caries properties, six characteristic features of the lesion were intendedly selected: *Depth, Length, Width, Ratio, Convex area, Smoothness*. However, it is impossible to calculate *Depth* from two-dimensional images of occlusal surfaces. Thus, for calculation, we only considered five features (*Length, Width, Ratio, Convex area, Smoothness*). The rectangular boundaries were drawn to cover the perimeter of the occlusal surface and the extracted carious lesion (see Figure 3). Inside this boundary of carious lesion, the *Length, Width,* and *Smoothness* of the lesion area were calculated. Then, based on boundary of the whole occlusal surface, the *Ratio* (ratio of carious lesion area to entire occlusal surface) and *Convex area* values could be determined. Finally, the table datasets for prediction models were formed by the obtained values of selected features (see Table 2).

#### 2.3.2. Classification Algorithms and Evaluation Metrics

After the values of features of carious lesion areas were calculated, we observed that all the values from tooth images were correctly identified as *“Non-Caries”*, equal to 0. The classification problem was now consequently reduced to only binary classification among the *“Caries”* category (VNC class versus C class). Prior to employing classification algorithms, the misidentified *“Caries”* images in the detection stage and whole images from the “*Non-Caries*” category were discarded from the dataset. The new binary labeled dataset retained a total of 462 images, in which VNC class—169, C class—293. Then, 169 images were randomly chosen from C class to make the same number of images as in VNC class to tackle the data imbalance.

To perform the classification task, five classifiers were employed: Support Vector Machine (SVM), Random Forests (RF), K-Nearest Neighbors (KNN), Gradient Boosted Tree (GBT), and Logistic Regression (LR). SVM [23] is a powerful binary classifier that was first proposed by Cortes and Vapnik. RF [24] is an ensemble learning method for classification, regression, and other tasks, consists of an ensemble of individual decision trees that are trained independently on a random subset of data. KNN [25] is a simple classification algorithm that searches through the entire training dataset for k-most similar instances when a prediction is required for an unseen data instance. GBT [26] is an empowerment of the decision tree that is the easiest and most intuitive algorithm in the literature while LR [27] is an extension of the linear regression model for classification problems with two possible outcomes. To avoid possibility of overfitting, 10-fold Cross-Validation was applied to evaluate models. The evaluation metrics employed in this study are Accuracy; Recall; Precision; F1-Score; Sensitivity; Specificity; and AUCROC (Area Under the Curve Receiver Operating Characteristic).

### 2.4. Convolutional Neural Network

In Section 2.3, feature selection and feature quantification were conducted to form the dataset that advantages the employment of five classifiers. However, to evaluate the effectiveness of the proposed feature engineering method, we have employed Convolutional Neural Networks (CNNs) with learned features extracted by CNNs themselves. In particular, the network architectures adopted in this paper are based on the ResNet [28] (ResNet-18, ResNet-50) and GoogleNet [29] architecture.

The original dataset is composed of 587 smartphone color images of 3 classes: NSC—73 images, VNC—220 images, *C*—294 images. The total images were randomly divided into 3 datasets: Test set—10% from each class; Validation set—10%; and Training set— remaining 80%. This experiment was conducted by both ResNet and GoogleNet architecture. The learning epoch was set as 10 and the learning rate was set at 10^−4^. The validation accuracy, test accuracy, and CPU time of both were recorded.

## 3. Results

### 3.1. Caries Detection Stage

Considering the *“Caries”* category as positive class and *“Non-Caries”* category as negative class, the detection stage in our method showed an accuracy of 87.39%, sensitivity of 89.88%, and specificity of 68.86% (see Table 3 for details). 

### 3.2. Caries Classification Stage

#### 3.2.1. Caries Classification with Selected Features

Results of the five predictive models were summarized in Table 4. SVM obtained the highest accuracy, recall, precision, F1-score, sensitivity, specificity, and AUCROC among the five implemented algorithms while LR obtained the lowest scores among them.

#### 3.2.2. Convolutional Neural Network Classification

The results of CNN models were reported in Table 5. It was observed that the validation accuracy and test accuracy of the GoogleNet model was the highest (71.67%, 65.52%) among three CNNs, and it also was the least time-consuming model (42 min for 10 epochs).

## 4. Discussion

In this study, we presented a two-stage computational system based on the processing of smartphone color images of unrestored extracted teeth for occlusal caries detection and classification. The accuracy, sensitivity, and specificity of this stage were 87.39%, 89.88%, and 68.86%, respectively, when compared to visual ranking using modified ICDAS criteria. As we have known, the visual-tactile examination is the most commonly used method for the diagnosis of dental caries. However, due to the differences in conditions during the examination and the disagreement in the diagnosis of dental caries by the examiners, the performance of the visual-tactile examination was substandard, with extensive variation in sensitivity scores while specificity scores were more stable [30,31]. Hence, our results demonstrated that the proposed method can automatically recognize carious lesion areas from digital images of unrestored extracted teeth with fair sensitivity and specificity.

The results of the experiments found clear support for the possibility of the caries detection system with input data as only color images of extracted teeth taken by smartphones, which certainly have lower quality than other digital camera images. Our methodology does not require any complex and expensive devices to acquire data since a common smartphone with a built-in camera can produce high-quality images. Although this method spectacularly identified the caries lesions in the *“Caries”* category, it could not totally detect minor lesions in the occlusal surfaces, especially in the VNC class (see Figure 5①). Owing to the presence of light reflection, several images were misidentified as *“Caries”* from the *“Non-Caries”* category (see Figure 5②) and it also affected recognizing correctly the lesion areas from the *“Caries”* category (see Figure 5③). In more detail, the shadow effects that appeared on the occlusal surfaces of the teeth possibly allowed the *“Non-Caries”* images to be misidentified as caries-like lesions, while the lesions areas from *“Caries”* images were identified as larger than they should be. Thence, in case carious lesions have failed to be detected by our proposed system, histological verification of the detection results needs to be performed to confirm the presence and extent of the lesions. Moreover, other confounding factors such as dental plaque, extrinsic stains, stained pits/fissures, and cracks on the occlusal surface have impacted the sensitivity of our system as well. Our proposed methodology has failed to detect carious lesion areas in case the dental plaque presents with an indistinguishable color spectrum from sound tooth areas (see Figure 5④). Likewise, as a consequence of the color similarity between extrinsic stains or stained pit/fissures and carious lesions that present in obtained images, our detection system could identify stains as caries-like lesions (see Figure 5⑤). The characteristic features of dental plaque and teeth stains need to be separately described as other inputs so that prediction models can correctly differentiate between them and true carious lesions. Unfortunately, this technical problem remains unsolved at the current stage of our study. We, therefore, have recommended that these confounding factors should be carefully cleaned out of teeth’s occlusal surfaces before taking photos. In addition, the performance of the caries detection system can be altered in case we intend to use the images of restored teeth with sealants, dental restorative materials, and even silver diamine fluoride (SDF) as input data. Associated with the presence of restorations on the occlusal surface, the failure of our system may occur in two cases: 1—if the color of dental restorations is nearly the same as the sound area, our system is able to identify them as “*Non-Caries*”; 2—if the color of dental restorations is dissimilar to the sound area, they probably are recognized as “*Caries*”. Overall, the results of our proposed method go beyond previous reports, showing that the sensitivity of our detection system was 89.88%, which was higher than the results of Kositbowornchai et al. [13] (77%) and Berdouses et al. [16] (80%) in terms of caries detection from digital images according to visual and image-based examination (see Table 6).

1. Minor change on the occlusal surface was not detected by the proposed method.
2. The proposed method recognized shadow effect that appeared on the occlusal surface of “*Non-Caries”* tooth as carious lesion (indicated by arrow).
3. The proposed method correctly recognized carious lesion on the occlusal surface, but due to light reflection, carious lesion was identified as larger than it should be.
4. The proposed method recognized shadow effect that appeared on the occlusal surface as carious lesion, while true lesion could not be identified due to appearance of dental plaque (indicated by arrow).
5. The proposed method misidentified carious lesion due to appearance of stains on occlusal surface (indicated by arrow).

In this paper, the feature selection was done by intendedly choosing features of carious lesion properties. The feature extraction and feature selection were keys of importance in machine learning, pattern recognition, and image processing. Olsen et al. [14] used the DASM (Directed Active Shape Modeling) algorithm to process color images, then the feature vector contains seven features extracted from the pixel: the magnitude of the gradient and six texture measures. Ghaedi et al. [15], after finishing feature extraction at 10 by 10 windows level and the whole image level, applied the information gain ratio method for feature selection. Then, the top 12 ranked features with 5 is the minimum number of features that were selected to use for caries classification. Berdouses et al. [16] extracted texture-based and intensity-based features from each pixel in the region of interest for a 15 by 15 neighborhood. After that, a correlation-based subset selection approach was applied for feature selection from 3 channels (Red, Green, and Blue) of color images, and as a result, 36 features were selected. The main thing in common with the above methods was that the pixel-based features were calculated from images and then feature selection was done by computational algorithms. In our case, after the detection stage, five characteristic features of the identified carious lesion areas were selected by experts and then quantified the values of these features. The main advantage of this approach is that these selected features were simple, easy to understand and have a strong relation to the clinical appearance of dental caries. However, the most important feature—the *Depth*, which could help to distinguish between early-stage and late-stage dental caries—could not be calculated from 2D images of occlusal surfaces.

With the dataset containing only quantified values of the five characteristic features, five different algorithms (SVM, RF, KNN, GBT, LR) were employed to classify between VNC class (Early-stage caries) and C class (Late-stage caries). Among these algorithms, it is clear that the highest performance was achieved when using the SVM predictive model. The accuracy, sensitivity, and specificity of this model were 88.76%, 92.31%, and 85.21%. The results now provide evidence of the feasibility of our proposed method. Our feature selection and quantification method provided meaningful input data to developing computational models for Early-stage caries classification. Compared with the best results which were achieved by using CNN models (GoogleNet model: validation accuracy—71.67% and test accuracy—65.52%), the accuracy of our model was higher and less time-consuming. For training CNN classification models, learned features are extracted by CNNs themselves. After setting up the training data, the CNN models train themselves and determine which input values are the most useful contributor for object detection. However, there is a possibility that the inside of the network may contain a number of insignificant predictor variables which the developer cannot simply determine. No well-established criteria can be used to interpret the weight in a connection weight matrix [32]. Besides, the number of data points in our study that we assigned was insufficient and imbalanced to train the CNN models which could predict reliably. Therefore, with our dataset, the proposed feature selection and quantification method helped to reduce the problem which may be caused by a small input dataset but still achieved robust results.

In general, moderately superior results are achieved with our proposed feature selection and quantification method. Unlike previous studies, we can precedently define specific features for ML algorithms in the dental caries classification task. The characteristic features, which clinically contributed to differentiate between different classes of carious lesions, were carefully selected based on standard knowledge by dental experts. These features are understandable for not only dental professionals but also computer scientists that could help to bridge the gap between the two disciplines. Moreover, since the quantified values of these features for each data point are truly small in size, the AI algorithms can process a huge amount of data in only a few minutes and do not require tremendous computational resources. In case we intend to integrate AI-based prediction models in a smartphone with limited computing capacity, this advantage of our methodology could play a crucial role in the operation process.

The limitations of the present study naturally include: the detection stage can be failed where the reflection of light is present; the presence of dental plaque, extrinsic stains, stained pits/fissures, and cracks on the occlusal surface might have impacted the sensitivity of detection system; the proposed method is unable to identify the depth of the cavity; dataset was only in vitro data (images of extracted teeth); the proposed method was based on previous extensive research findings, and histological verification of the diagnosis has not been conducted to confirm the presence and extent of the lesions. Hence, gold standard methods such as histological verification, Micro CT, or Polarized Light Microscopy are expected to be performed in future work to validate the effectiveness of the proposed computational system in detecting and classifying carious lesions on occlusal surfaces of extracted teeth. Furthermore, the major challenges in continuing technology development are finding trustworthy methods to taking intra-oral photos of posterior teeth and to examine the margins of dental restorations on the teeth’s occlusal surfaces. To tackle these issues, future research should be conducted with more realistic data to widely investigate the possibility of the proposed method in clinical settings. With a focus on collecting informative in vivo data, an appropriate system for accurately taking intra-oral images that can capture entire dental arches including the occlusal surfaces of premolars and molars needs to be developed. However, to implement our method with in vivo data from patients, several important issues should be considered. The algorithms which can be used for tooth annotation and then segmenting the images of each tooth from the dental arch to analyze its properties are necessary.

## 5. Conclusions

In conclusion, we developed a two-stage computational system that can detect early occlusal caries from smartphone color images of unrestored extracted teeth: in the first stage, carious lesion areas were identified and extracted from sound tooth regions, then five characteristic features of these areas were intendedly selected and calculated to be inputted into the classification stage. The performance of the proposed system in classifying occlusal caries is comparable with the visual examination given by dentists. The designed method is non-invasive, comparatively inexpensive, not requesting large computational resources, less time-consuming, and simple to implement, especially in public health investigations. Although the performance of the proposed method is promising, more research is needed to apply and test with in vivo data in future investigations.

## Figures and Tables

**Figure 5:**
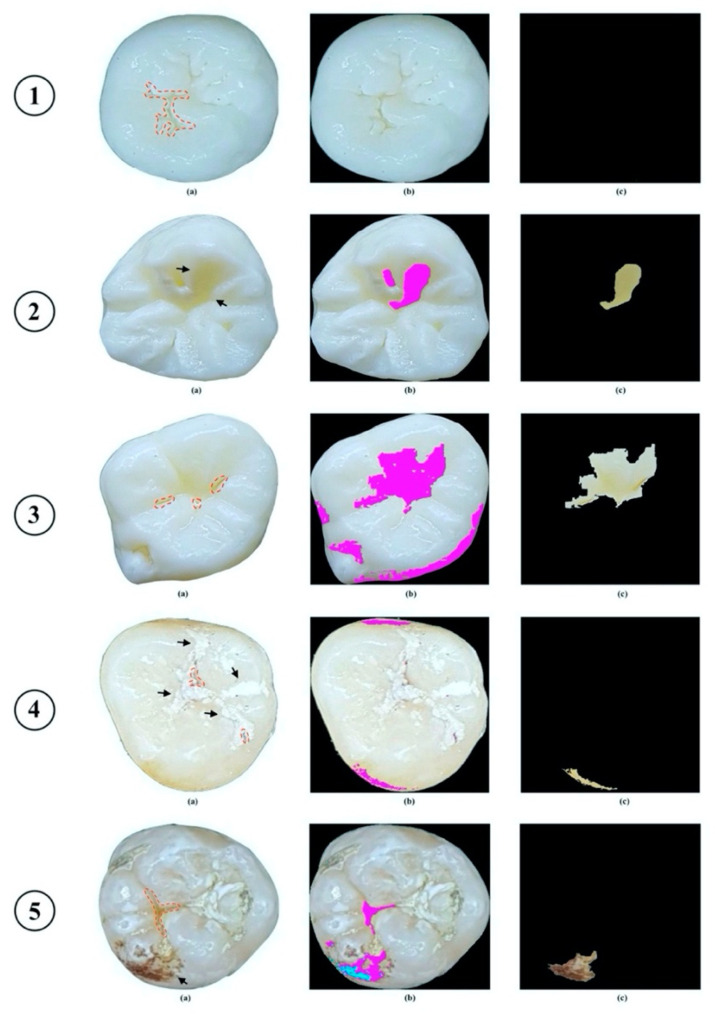
Examples of failures on caries detection (presence of confounding factors on occlusal surfaces were indicated by black arrows). (**a**) Carious lesion area was identified by the experts; (**b**) carious lesion area was identified by proposed method; (**c**) extracted carious lesion area.

**Table 1:** Data attributes.

	ICDAS Criteria Code						
	*0*	*1*	*2*	*3*	*4*	*5*	*6*
**Labeled classes**	No Surface Change (NSC) (*n* = 73)	Visually Non-Cavitated (VNC) (*n* = 220)		Cavitated (C) (*n* = 294)			

**Table 2:** Features used for building the proposed predictive models.

	Features of Carious Lesions				
	Length	Width	Smoothness	Ratio	Convex Area
**NSC**	0	0	0	0	0
**VNC**	✓	✓	✓	✓	✓
**C**	✓	✓	✓	✓	✓

“✓” symbol defines the existence of values in the classes.

**Table 3:** Confusion matrix for caries detection stage.

	True Class		
**Predicted** **class**		**Caries**	**Non-Caries**
	**Caries**	462	22
	**Non-Caries**	52	51

**Table 4:** Performance in the prediction of the employed classifiers.

	Accuracy (%)	Recall (%)	Precision (%)	F1 Score (%)	Sensitivity (%)	Specificity (%)	AUCROC (%)
***SVM***	88.76	92.31	86.19	89.14	92.31	85.21	95
***RF***	86.39	87.57	85.55	86.55	87.57	85.21	94
***KNN***	86.09	86.98	85.47	86.22	86.98	85.21	94
***GBT***	85.21	85.80	84.80	85.29	85.80	84.62	92
***LR***	82.25	84.62	80.79	82.66	84.62	79.88	92

**Table 5:** Results of CNN classification experiment.

	Validation Accuracy (%)	Test Accuracy (%)	Learning Epochs	CPU Time (m)
***ResNet*** ***18***	71.67	51.72	10	50
***ResNet*** ***50***	68.33	63.79	10	94
***GoogleNet***	71.67	65.52	10	42

**Table 6:** The detection performance of the proposed method compares with other studies in the literature.

Authors	Methodology	Dataset	Accuracy	Sensitivity	Specificity
Kositbowornchai et al. [13]	Images from a charged coupled device (CCD) camera and intra-oral digital radiography	*Training set*—49 images (26 teeth were sound or had artificially-created buccal or lingual carious lesions; 23 teeth with sound or artificially-induced proximal caries) *Test set*—322 images (160 CCD images and 162 digital radiographs)		CCD: 77% Radiograph: 81%	CCD: 85% Radiograph: 93%
Berdouses et al. [16]	Digital color images Preprocessing Segmentation Postprocessing	103 images: 12 in vivo; 91 in vitro.	80%	80%	
Our method	Smartphone color images Image Processing	587 in vitro images	87.39%	89.88%	68.86%

## Data Availability

The data presented in this study are available on request from the corresponding author.

## References

[1] Global Burden of Disease Study C. (2015). Global, regional, and national incidence, prevalence, and years lived with disability for 301 acute and chronic diseases and injuries in 188 countries, 1990-2013: A systematic analysis for the Global Burden of Disease Study 2013. Lancet.

[2] Neuhaus K.W., Ellwood R., Lussi A., Pitts N.B. (2009). Traditional lesion detection aids. Monogr. Oral. Sci..

[3] Amaechi B.T., Podoleanu A., Higham S.M., Jackson D.A. (2003). Correlation of quantitative light-induced fluorescence and optical coherence tomography applied for detection and quantification of early dental caries. J. Biomed. Opt..

[4] Abrams S.H., Sivagurunathan K.S., Silvertown J.D., Wong B., Hellen A., Mandelis A., Hellen W.M., Elman G.I., Mathew S., Mensinkai P.K. (2017). Correlation with caries lesion depth of the Canary System, DIAGNODENT and ICDAS II. Open Dent. J..

[5] Jeon R.J., Matvienko A., Mandelis A., Abrams S.H., Amaechi B.T., Kulkarni G. (2007). Detection of interproximal demineralized lesions on human teeth in vitro using frequency-domain infrared photothermal radiometry and modulated luminescence. J. Biomed. Opt..

[6] Attrill D.C., Ashley P.F. (2001). Occlusal caries detection in primary teeth: A comparison of DIAGNOdent with conventional methods. Br Dent J.

[7] Söchtig F., Hickel R., Kühnisch J. (2014). Caries detection and diagnostics with near-infrared light transillumination: Clinical experiences. Quintessence Int..

[8] Davies G.M., Worthington H.V., Clarkson J.E., Thomas P., Davies R.M. (2001). The use of fibre-optic transillumination in general dental practice. Br. Dent. J..

[9] Ricketts D.N., Kidd E.A., Liepins P.J., Wilson R.F. (1996). Histological validation of electrical resistance measurements in the diagnosis of occlusal caries. Caries Res..

[10] Bottenberg P., Jacquet W., Behrens C., Stachniss V., Jablonski-Momeni A. (2016). Comparison of occlusal caries detection using the ICDAS criteria on extracted teeth or their photographs. BMC Oral Health.

[11] Boye U., Walsh T., Pretty I.A., Tickle M. (2012). Comparison of photographic and visual assessment of occlusal caries with histology as the reference standard. BMC Oral Health.

[12] Umemori S., Tonami K., Nitta H., Mataki S., Araki K. (2010). The possibility of digital imaging in the diagnosis of occlusal caries. Int. J. Dent..

[13] Kositbowornchai S., Siriteptawee S., Plermkamon S., Bureerat S., Chetchotsak D. (2006). An Artificial Neural Network for Detection of Simulated Dental Caries. Int. J. Comput. Assist. Radiol. Surg..

[14] Olsen G. (2010). Fundamental Work toward an image Processing-Empowered Dental Intelligent Educational System. Ph.D. Thesis.

[15] Ghaedi L., Gottlieb R., Sarrett D.C., Ismail A., Belle A., Najarian K., Hargraves R.H. (2014). An automated dental caries detection and scoring system for optical images of tooth occlusal surface. Conf Proc IEEE Eng Med Biol Soc.

[16] Berdouses E.D., Koutsouri G.D., Tripoliti E.E., Matsopoulos G.K., Oulis C.J., Fotiadis D.I. (2015). A computer-aided automated methodology for the detection and classification of occlusal caries from photographic color images. Comput. Biol. Med..

[17] Ahmad I. (2009). Digital dental photography. Part 4: Choosing a camera. Br. Dent. J..

[18] Kohara E.K., Abdala C.G., Novaes T.F., Braga M.M., Haddad A.E., Mendes F.M. (2018). Is it feasible to use smartphone images to perform telediagnosis of different stages of occlusal caries lesions?. PLoS ONE.

[19] Estai M., Kanagasingam Y., Huang B., Shiikha J., Kruger E., Bunt S., Tennant M. (2017). Comparison of a Smartphone-Based Photographic Method with Face-to-Face Caries Assessment: A Mobile Teledentistry Model. Telemed J. E Health.

[20] Banting D., Eggertsson H., Ekstrand K., Ferreira-Zandoná A., Ismail A., Longbottom C., Pitts N., Reich E., Ricketts D., Selwitz R. (2005). Rationale and evidence for the international caries detection and assessment system (ICDAS II). Ann. Arbor..

[21] Duong D.L., Kabir M.H., Kuo R.F. (2021). Automated caries detection with smartphone color photography using machine learning. Health Inform. J..

[22] Bradski G. (2000). The opencv library. Dr Dobb’s J. Softw. Tools.

[23] Cortes C., Vapnik V. (1995). Support-vector networks. Mach. Learn..

[24] Breiman L. (2001). Random forests. Mach. Learn..

[25] Keller J., Gray M.R., Givens J.A. (1985). A fuzzy K-nearest neighbor algorithm. IEEE Trans. Syst. Man Cybern..

[26] Friedman J.H. (2001). Greedy function approximation: A gradient boosting machine. Ann. Stat..

[27] Hastie T., Tibshirani R., Friedman J. (2009). The Elements of Statistical Learning.

[28] He K., Zhang X., Ren S., Sun J. Deep Residual Learning for Image Recognition. Proceedings of the 2016 IEEE Conference on Computer Vision and Pattern Recognition (CVPR).

[29] Szegedy C., Wei L., Yangqing J., Sermanet P., Reed S., Anguelov D., Erhan D., Vanhoucke V., Rabinovich A. Going deeper with convolutions. Proceedings of the 2015 IEEE Conference on Computer Vision and Pattern Recognition (CVPR).

[30] Bader J.D., Shugars D.A., Bonito A.J. (2001). Systematic reviews of selected dental caries diagnostic and management methods. J. Dent. Educ..

[31] Bader J.D., Shugars D.A., Bonito A.J. (2002). A systematic review of the performance of methods for identifying carious lesions. J. Public. Health Dent..

[32] Tu J.V. (1996). Advantages and disadvantages of using artificial neural networks versus logistic regression for predicting medical outcomes. J. Clin. Epidemiol..

